# Synthesis and Cytotoxicity Evaluation of Novel Asymmetrical Mono-Carbonyl Analogs of Curcumin (AMACs) against Vero, HeLa, and MCF7 Cell Lines

**DOI:** 10.3390/scipharm86020025

**Published:** 2018-06-07

**Authors:** Pekik Wiji Prasetyaningrum, Anton Bahtiar, Hayun Hayun

**Affiliations:** Faculty of Pharmacy, Universitas Indonesia, Depok 16424, West Java, Indonesia; pekikwiji@gmail.com (P.W.P.); anton.bahtiar@gmail.com (A.B.)

**Keywords:** asymmetrical mono-carbonyl analogs of curcumin, AMACs, synthesis, cytotoxicity, Vero, HeLa, MCF7, cell lines

## Abstract

A series of novel asymmetrical mono-carbonyl analogs of curcumin (AMACs) were synthesized and evaluated for cytotoxic activity using BSLT and MTT assay against Vero, HeLa, and MCF7 cell lines. The structures of the synthesized compounds were confirmed by FTIR, ^1^H-NMR, ^13^C-NMR, and mass spectral data. The results of the cytotoxicity evaluation showed that the synthesized compounds exhibited moderate to very high toxic activity in BSLT (LC_50_ value 29.80–1704.23 µM); most of the compound exhibited cytotoxic activity against HeLa cell lines, which is comparable to the activity of cisplatin (IC_50_ value 40.65–95.55 µM), and most of the compound tested against MCF7 cell lines exhibited moderate to very high cytotoxic activity (IC_50_ value 7.86–35.88 µM). However, the selectivity index (SI) of the compounds was low (<1–1.96). Among the synthesized compounds, compound **1b** was the most cytotoxic and selective against MCF7 cell lines. It could be considered for further development to obtain the more active and selective chemotherapeutic agents against breast cancer.

## 1. Introduction

Cancer is one of the main causes of death worldwide, especially in developing countries. Breast and cervix uterine cancer have the highest cancer incidence in Indonesian female populations with 48,998 and 20,928 cases, respectively. The cancer mortality pofile reported in 2014 showed that the mortality caused by the two cancers were 21.4% and 10.3% [1]. For many years, chemotherapeutic agents have been developed and used to treat cancer. Unfortunately, there is no drug shows good selectivity for cancer cells. Many chemotherapeutic drugs produce serious chronic and delayed toxicities that may be irreversible, particularly in the heart, lungs, and kidneys [2]. Curcumin (diferuloylmethane) demonstrated various biological activities such as growth suppression in a wide variety of tumor cells, as well as chemopreventive effects on certain types of cancers with low toxicity [3]. Nevertheless, it has not yet been accepted as a therapeutic compound because of its low chemical stability, low solubility, poor absorption, and rapid metabolism, resulting in low bioavailability and weak in vivo biological activity [4,5,6,7]. Many curcumin analogs have been synthesized and investigated, such as mono-carbonyl analogs of curcumin (MACs), to improve its bioactivity, stability, and bioavailability.

**Scheme 1:**
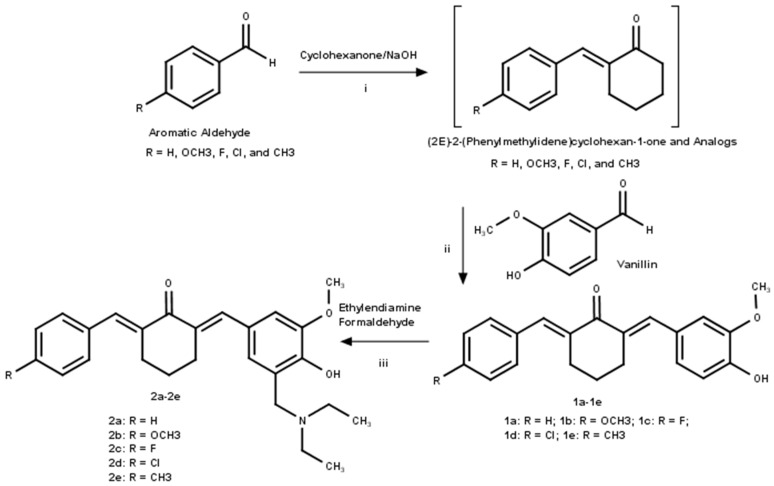
Synthesis of the title compounds (**1a**–**1e** and **2a**–**2e**). Conditions: (i) r.t., 2 h.; (ii) ethanol, reflux, diluted HCl/ethanol, 30 min.; and (iii) ethanol, reflux, 7–11 h.

The mono-carbonyl analogs of curcumin (MACs) exhibited a potency of 10–30 times for cell lines and cellular proteins compared to curcumin [8,9,10,11]. Some MACs compounds with acetone and cyclohexanone as a linker between the two phenyl rings inhibited the growth of leukemia, colon, renal, melanoma, ovarian, central nervous system (CNS), and prostate cancer cells better than cisplatin [12]. The MACs pharmacokinetic profile is much more stable than curcumin, resulting in higher tumor regression [8,12]. Nowadays, some of the asymmetrical mono-carbonyl analogs of curcumin (AMACs) with different constituents on the two phenyl rings have been developed and reported to show antioxidant, anti-inflammatory, antimicrobial [13,14,15,16,17], and antitumor properties [18]. However, reports on studies of AMACs compounds as anti-cancer agents are still limited. To further explore AMACs as anticancer compounds, we report the synthesis and in vitro cytotoxicity evaluation of novel AMACs (**1a**–**1e** and **2a**–**2e**, Scheme 1) against Vero, HeLa, and MCF7 Cell lines.

## 2. Materials and Methods

### 2.1. Chemistry

#### 2.1.1. General Procedures

All solvents, chemicals, and reagents were obtained commercially and used without purification. Purity tests of the products were performed using thin layer chromatographic (TLC) method on silica gel 60 F254 plates (Merck, Darmstadt, Germany). Melting points were determined in the capillary tube using melting point apparatus (Stuart Scientific, Bibby Sterilin, Staffordshire, UK) and are uncorrected. Infrared (IR) spectra were recorded on an FTIR 8400S spectrophotometer (Shimadzu, Kyoto, Japan). ^1^H-NMR and ^13^C-NMR spectra were recorded on Nuclear Magnetic Resonance (NMR) spectrometer (Agilent, Santa Clara, CA, USA) at 500 MHz for 1H and 125 MHz for ^13^C using tetramethylsilane (TMS) as internal standard, and High-resolution mass spectra (HRMS) were measured with a Waters LCT Premier XE (ESI-TOF) (Waters Corp., Milford, MA, USA) system in negative mode.

#### 2.1.2. Synthesis of (2E)-2-(phenylmethylidene)cyclohexan-1-one and Analogs

The syntheses were performed according to the synthesis method used for 2-benzylidene acetone by replacing acetone with cyclohexanone [19]. A mixture of aromatic aldehyde (0.32 mol) and cyclohexanone (0.88 mol) was added to a solution of NaOH (10%) dropwise while stirring for 2 h. The mixture was neutralized with dilute HCl to pH 7, the organic layer was separated, and the water layer was extracted with 16 mL of toluene. The toluene layer was mixed with the organic layer, washed with 16 mL of water, dried with anhydrous sodium sulfate, and evaporated using rotary vacuum evaporator to give the crude product. The crude product was used as the starting material for the next step without further purification.

#### 2.1.3. Synthesis of Asymmetrical Mono-Carbonyl Analogs of Curcumin (AMACs) (**1a**–**1e**)

The synthesis of the compounds was performed by aldol condensation of (2E)-2-(phenylmethylidene)cyclohexan-1-one or its analogs and vanillin under acidic condition. The mixture of (2E)-2-(phenylmethylidene)cyclohexan-1-one or its analogs (0.005 mol) and vanillin (0.01 mol) in ethanol (10 mL) was heated under reflux condition until dissolved and a drop of diluted HCl/ethanol (1 drop: 1 mL) was added and stirred for 30 mins. The progress of the reaction was monitored using TLC method. Upon completion, the solvent was evaporated, and the solid material obtained was triturated with a cold mixture of glacial acetic acid/water (1:1) and filtered using the Buchner funnel. The solid product obtained was washed with a cold mixture of glacial acetic acid/water (1:1), dried, and purified by column chromatography with a mixture of the appropriate ratio of n-hexane and ethyl acetate.

*(2E,6E)-2-[(4-hydroxy-3-methoxyphenyl)methylidene]-6-(phenylmethylidene)cyclohexan-1-one* (**1a**). The compound was a bright yellow powder, in a 50.0% yield, m.p.: 149–151 °C and Rf = 0.8 (ethyl acetate: n-hexane = 1:2). FTIR (KBr) υmax cm^−1^: 3211 (OH), 2999 (CH aromatic), 2837 (CH aliphatic), 1647 (C=O), 1587, 1531, 1448 (C=C), 1174 (C-O). ^1^H-NMR (500 MHz, CDCl_3_), δ/ppm: 1.80 (m, 2H, C-CH_2_-C_cyclohexanone_); 2.93 (m, 4H, =C-CH_2_-C_cyclohexanone_); 3.91 (s, 3H, OCH_3_); 5.89 (s, 1H, OH); 6.95 (d, 1H, *J* = 8.5, H_Ar_); 7.00 (s, 1H, H_Ar_; 7.09 (d, 1H, *J* = 8.3 Hz, H_Ar_); 7.33 (t, 1H, *J* = 7.4, H_Ar_); 7.39 (t, 2H, *J* = 7.3 Hz, H_Ar_); 7.46 (d, 2H, *J* = 7.2 Hz, H_Ar_); 7.75 and 7.79 (s, 1H Ar-CH=C and 1H, C=CH-Ar). ^13^C-NMR (100 MHz, CDCl_3_) δ/ppm: 23.1 (1C, C-CH_2_-C_cyclohexanone_), 28.5 (1C, =C-CH_2_-C_cyclohexanone_), 28.7 (1C, =C-CH_2_-C_cyclohexanone_), 56.0 (O-CH_3_), 113.4, 114.5, 124.6, 128.6, 130.4, 134.2 (10C, C_Ar_), 136.1, 137.5 (4C, -C=C-) 146.4 (2C, C_Ar_-O), 190.3 (1C, C=O) [20]. HRESIMS (**m*/*z*)* found 319.1346 ([[M − H]]^−^) calculated masses for C_21_H_19_O_3_: 319.1334.

*(2E,6E)-2-[(4-hydroxy-3-methoxyphenyl)methylidene]-6-[(4-methoxyphenyl)methylidene] cyclohexan-1-one* (**1b**). The compound was a yellow powder, in a 2.7% yield, m.p.: 133–136 °C and Rf = 0.55 (ethyl acetate: n-hexane = 1:2). FTIR (KBr) υmax cm^−1^: 3431 (OH), 3003 (CH aromatic), 2935 (CH aliphatic), 1734 (C=O), 1656, 1593 and 1512 (C=C), 1161 (C-O). ^1^H NMR (500 MHz, CDCl_3_), δ/ppm: 1.80 (m, 2H, C-CH_2_-C_cyclohexanone_); 2.92 (m, 4H, =C-CH_2_-C_cyclohexanone_); 3.84 (s, 3H, CH_3_-O); 3,91 (s, 3H, CH_3_-O); 5.86 (s, 1H, OH); 6.92 (d, 1H, *J* = 8.5, H_Ar_); 6.99 (s, 1H, H_Ar_); 7.08 (d, 1H, *J* = 8.3 Hz, H_Ar_); 7.46 (d, 2H, *J* = 8.3, Hz, H_Ar_); 7.73 and 7.76 (s, 1H, Ar-CH=C and s, 1H, C=CH-Ar). ^13^C-NMR (100 MHz, CDCl_3_) δ/ppm: 23.1 (1C, C-CH_2_-C_cyclohexanone_), 28.5 (2C, =C-CH_2_-C_cyclohexanone_), 56.0 (1C, OCH_3_), 55.4 (1C, OCH_3_), 113.3, 114.4, 124.5, 128.5, 132.3 (9C, C_Ar_), 134.4, 137.0 (4C, -C=C-), 146.4,146.5,160.0 (3C, C_Ar_-O) 190.1 (1C, C=O) [20]. HRESIMS (**m*/*z*)* found 349.1432 ([[M − H]]^−^) calculated masses for C_22_H_21_O_4_ : 349.1440.

*(2E,6E)-2-[(4-hydroxy-3-methoxyphenyl)methylidene]-6-[(4-fluorophenyl)methylidene] cyclohexan-1-one* (**1c**). The compound was a light yellow powder, in a 47.3% yield, m.p.: 129–131 °C and Rf = 0.52 (ethyl acetate : n-hexane = 1:2). FTIR (KBr) υmax cm^−1^: 3313 (OH), 3003 (CH aromatic), 2939 (CH aliphatic), 1734 (C=O), 1656, 1604 and 1514 (C=C), 1220 (C-F), 1155 (C-O). ^1^H-NMR (500 MHz, CDCl_3_), δ/ppm: 1.82 (m, 2H, C-CH_2_-C_cyclohexanone_); 2.89 (t, 2H, *J* = 7.1 Hz, =C-CH_2_-C_cyclohexanone_); 2.93 (t, 2H, *J* = 6.1 Hz, =C-CH_2_-C_cyclohexanone_); 3.93 (s, 3H, CH_3_-O); 5.90 (s, 1H, -OH); 6.96 (d, 1H, *J* = 9.3 Hz, H_Ar_); 7.00 (s, 1H, H_Ar_); 7.11 (d, 3H, *J* = 8.2 Hz, H_Ar_); 7.44 (dd, 2H, *J* = 5.5 Hz, H_Ar_); 7.75 (s, 2H, Ar-CH=C). ^13^C NMR (100 MHz, CDCl_3_) δ/ppm: 23.1 (1C, C-CH_2_-C_cyclohexanone_), 28.7 (2C, =C-CH_2_-C_cyclohexanone_), 56.0 (1C, OCH_3_), 113.4, 114.5, 115.5, 115.6, 124.6, 128.5, 132.3, 134.1 (9C, C_Ar_), 135.5, 136.0, 137.8, 146.4 (4C, C=C), 163.7 (C_Ar_-F), 146.7, 160.7 (2C, C_Ar_-O), 190.1 (1C, C=O) [20]. HRESIMS (**m*/*z*)* found 337.1270 ([[M − H]]^−^) calculated masses for C_21_H_18_FO_3_ : 337.1240.

*(2E,6E)-2-[(4-hydroxy-3-methoxyphenyl)methylidene]-6-[(4-chlorophenyl)methylidene] cyclohexan-1-one* (**1d**). The compound was a light yellow powder, in a 6.1% yield, m.p.: 150–154 °C and Rf = 0.75 (ethyl acetate:n-hexane = 1:2). FTIR (KBr) υmax cm^−1^: 3296 (OH), 3003 (CH aromatic), 2939 (CH aliphatic), 1734 (C=O), 1658, 1604 and 1514 (C=C), 1163 (C-O), 833 (C-Cl). ^1^H-NMR (500 MHz, CDCl_3_), δ/ppm: 1.80 (m, 2H, C-CH_2_-C_cyclohexanone_); 2.87 (t, 2H, *J* = 7.3 Hz, =C-CH_2_-C_cyclohexanone_); 2.93 (t, 2H, *J* = 7.3 Hz, =C-CH_2_-C_cyclohexanone_); 3.92 (s, 3H, CH_3_-O); 5.86 (s, 1H, OH); 6.96 (d, 1H, *J* = 8 2 Hz, H_Ar_); 6.99 (s, 1H, H_Ar_); 7.08 (d, 1H, *J* = 8.2 Hz, H_Ar_); 7.37 (d, 2H, *J* = 8.8 Hz, H_Ar_); 7,39 (d, 2H, *J* = 9.3 Hz, H_Ar_), 7.72, 7.74 (s, 1H, Ar-CH=C and s, 1H, C=CH-Ar). ^13^C-NMR (100 MHz, CDCl_3_) δ/ppm: 23.0 (1C, C-CH_2_-C_cyclohexanone_), 28.5, 28.6 (2C, =C-CH_2_-C_cyclohexanone_), 56.1 (1C, OCH_3_), 113.4, 114.5, 124.7, 128.5 (C15), 129.2, 131.6., 134.1 (9C, C_Ar_), 134.6, 135.2, 136.8, 137.8 (4C, -C=C-), 134.0. (C_Ar_-Cl), 146.6, 146.7 (2C, C_Ar_-O), 196.1 (1C, C=O) [20]. HRESIMS (**m*/*z*)* found 353.0947 ([[M − H]]^−^) calculated masses for C_21_H_18_ClO_3_ : 353.0945.

*(2E,6E)-2-[(4-hydroxy-3-methoxyphenyl)methylidene]-6-[(4-methylphenyl)methylidene] cyclohexan-1-one* (**1e**). The compound was a yellow powder, in a 13.2% yield, m.p.: 130–131 °C and Rf = 0.75 (ethyl acetate : n-hexane = 1:2). FTIR (KBr) υmax cm^−1^: 3323 (OH), 3007 (CH aromatic), 2939 (CH aliphatic), 1734 (C=O), 1653, 1593 and 1462 (C=C), 1161 (C-O). ^1^H-NMR (500 MHz, CDCl_3_), δ/ppm: 1.79 (m, 2H, C-CH_2_-C_cyclohexanone_); 2.38 (s, 3H, CH_3_-Ar), 2.91 (t, 2H, *J* = 5.0 Hz, =C-CH_2_-C_cyclohexanone_); 2.93 (t, 2H, *J* = 5.0 Hz, =C-CH_2_-C_cyclohexanone_), 3.92 (s, 3H, CH_3_-O); 5.86 (s, 1H, -OH); 6.96 (t, 3H, *J* = 8.2 Hz, H_Ar_); 6.99 (s, 1H, H_Ar_); 7.08 (d, 1H, *J* = 8.3 Hz, C15=CH-C18)); 7.22 (d, 2H, *J* = 9.0 Hz, H_Ar_); 7.37 (d, 2H, *J* = 9.0 Hz, H_Ar_), 7,74 and 7.77 (s, 1H, s, 1H, Ar-CH=C and s, 1H, C=CH-Ar). ^13^C-NMR (100 MHz, CDCl_3_) δ/ppm: 21.5 (1C, CH_3_-Ar), 23.1 (1C, C-CH_2_-C_cyclohexanone_), 28.6, 28.7 (2C, =C-CH_2_-C_cyclohexanone_), 56.0 (CH_3_-O), 113.4, 114.5, 124.6, 128.6, 129.2, 130.5, 133.3, 134.3 (10C, C_Ar_), 134.6, 135.2, 136.8, 137.8 (4C, -C=C-), 146.4, 146.5 (2C, C_Ar_-O), 190.3 (1C, C=O) [20]. HRESIMS (**m*/*z*)* found 333.1492 ([[M − H]]^−^) calculated masses for C_22_H_21_O_3_: 333.1491.

#### 2.1.4. Synthesis of Diethylamine Mannich Base of AMACs (**2a–2e**)

The syntheses were performed according to the method used for the synthesis of di-Mannich bases of curcumin and the synthesis of 2-[(2,6-dimethylmorpholin-4-yl)methyl]-4-[(E)-2-{3[(E)-2-{3- [(2,6-dimethylmorpholin-4-yl)methyl]-4-hydroxy-5-methoxyphenyl}ethenyl]-1H-pyrazol-5yl}etheyl]-6-methoxyphenol reported previously [21,22]. Compound **1a–1e** (2 mmol) separately were dissolved in ethanol, cooled in an ice bath, and diethylamine (5–7 mmol) and formaldehyde solution 37% (5–7 mmol) were added slowly. The mixture was stirred for 30 min at room temperature and then refluxed for 7–11 h. The progress of the reaction was monitored by TLC. After the reaction was completed, the solvent was evaporated to obtain the solid residue. The residue was dissolved in methanol (40 mL) and evaporated to a residue. The residue was then dissolved in methanol (50 mL), warmed, and poured slowly with constant stirring into about 400 mL of cold distilled water. The solvent was decanted, and the precipitate obtained was filtered off, washed with cold distilled water, dried at room temperature, and then purified by column chromatography.

*(2E,6E)-2-({3-[(diethylamino)methyl]-4-hydroxy-5-methoxyphenyl}methylidene)-6-(phenylmethylidene)cyclohexan-1-one* (**2a**). The compound was a caramel-like solid, in a 65.5% yield, m.p.: 79–80 °C and Rf = 0.51 (ethyl acetate: ethanol = 1:1). FTIR (KBr) υmax cm^−1^: 3053 (CH aromatic), 2972 (C-H), 1660 (C=O), 1599 (C=C), 1269 (C-N), 1157 (C-O). ^1^H-NMR (500 MHz, CD_3_OD), δ/ppm: 1.18 (t, 6H, *J* = 7.2 Hz, CH_3_-CH_2_-), 1.77 (m, 2H, C-CH_2_-C_cyclohexanone_); 2.77 (q, 4H, *J* = 7.0 Hz, CH_3_-CH_2_-N), 2.89 and 2.94 (t, 4H, *J* = 7.0 Hz, =C-CH_2_-C_cyclohexanone_), 3.85 (s, 3H, CH_3_-O), 3.95 (s, 2H, Ar-CH_2_-N), 6.92 (s, 1H, H_Ar_), 7.04 (s, 1H, H_Ar_); 7.33 (t, 1H, *J* = 8,7 Hz, H_Ar_); 7.40 (t, 2H, *J* = 7.4 Hz H_Ar_), 7.45 (d, 2H, *J* = 10.9 Hz, H_Ar_), 7.66 and 7.68 (s, 1H, s, 1H, Ar-CH=C and s, 1H, C=CH-Ar). ^13^C-NMR (100 MHz, CD_3_OD) δ/ppm: 10.9 (2C, CH_3_-CH_2_) 24.1 (1C, C-CH_2_-C_cyclohexanone_), 29.3 and 29.9 (2C, C-CH_2_-C_cyclohexanone_), 47.5 (1C, CH_3_-CH_2_-N-), 56.9 (1C, Ar-CH_2_-N), 56.4 (1C, CH_3_-O), 114.9, 122.2, 126.6, 126.4. 129.5, 129.7, 131.3, 133.9 (10C, C_Ar_), 137.2, 137.3, 137.9, 139.7 (4C, -C=C-), 149,7 and 153.0 (2C, C_Ar_-O), 191.8 (1C, C=O) [20] HRESIMS (**m*/*z*)* found 404.2285 ([[M − H]]^−^) calculated masses for C_26_H_30_NO_3_: 404.2226.

*(2E,6E)-2-({3-[(diethylamino)methyl]-4-hydroxy-5-methoxyphenyl}methylidene)-6-[(4-methoxyphenyl)methylidene]cyclohexan-1-one* (**2b**). The compound was an orange sticky powder, in a 46.8% yield, m.p.: 98–99 °C and Rf = 0.51 (ethyl acetate: ethanol = 1:1). FTIR (KBr) υmax cm^−1^: 3059 (CH aromatic), 2970 (CH aliphatic), 1734 (C=O), 1556 (C=C), 1595 and 1510 (C=C aromatic) 1271 (C-N), 1155 (C-O). ^1^H NMR (500 MHz, CD_3_OD), δ/ppm: 1.17 (t, 6H, *J* = 7.1, CH_3_-CH_2_-), 1.78 (m, 2H, C-CH_2_-C_cyclohexanone_); 2.77 (q, 4H, *J* = 7.1 Hz, CH_3_-CH_2_-N), 2.88 and 2.92 (t, 2H, *J* = 5.4 Hz, =C-CH_2_-C_cyclohexanone_ and t, 2H, *J* = 5.4 Hz, =C-CH_2_-C_cyclohexanone_), 3.81 (s, 3H, CH_3_-O-), 3.85 (s, 3H, CH_3_-O-), 3.91 (s, 2H, Ar-CH_2_-N), 6.89 (s, 1H, H_Ar_), 6.96 (d, 2H, *J* = 8.2, H_Ar_), 7.01 (s, 1H, H_Ar_); 7.44 (d, 2H, *J* = 8.8 Hz, H_Ar_), 7.64 and 7.65 (s, 1H, Ar-CH=C and s, 1H, C=CH-Ar). ^13^C NMR (100 MHz, CD_3_OD) δ/ppm: 11.0 (2C, CH_3_-CH_2_) 24.1 (1C, C-CH_2_-C_cyclohexanone_), 29.4 and 29.6 (2C, C-CH_2_-C_cyclohexanone_), 47.4 (2C, CH_3_-CH_2_-N-), 56.3 (1C, CH_3_-O), 55.8 (1C, CH_3_-O), 57.0 (1C, Ar-CH_2_-N), 114.8, 122.8, 126.7, 126.2, 126.7, 133.4 (9C, C_Ar_), 129.7, 135.6, 137.7, 139.3 (4C, -C=C-), 149.6, 152.8 and 161.7 (3C, C_Ar_-O), 191.9 (1C, C=O) [20]. HRESIMS (*m*/*z*) found 434.2101 ([[M − H]]^−^) calculated masses for C_27_H_32_NO_4_ : 434.2332.

*(2E,6E)-2-({3-[(diethylamino)methyl]-4-hydroxy-5-methoxyphenyl}methylidene)-6-[(4-fluorophenyl)methylidene]cyclohexan-1-one* (**2c**). The compound was an orange powder, in a 33.01% yield, m.p.: 79–81 °C and Rf = 0.48 (ethyl acetate: ethanol = 1:1). FTIR (KBr) υmax cm^−1^: 3041 (CH aromatic), 2937 (CH aliphatic), 1734 (C=O), 1656 (C=C), 1595 and 1492 (C=C aromatic) 1271 (C-N),1224 (C-F), 1157 (C-O). ^1^H-NMR (500 MHz, CD_3_OD), δ/ppm: 1.18 (t, 6H, *J* = 7.2 Hz, CH_3_-CH_2_-), 1.77 (m, 2H, C-CH_2_-C_cyclohexanone_); 2.77 (q, 4H, *J* = 7.2 Hz, CH_3_-CH_2_-N), 2.87 and 2.94 (t, 2H, *J* = 5.5 Hz, =C-CH_2_-C_cyclohexanone_ and t, 2H, *J* = 5.2 Hz, =C-CH_2_-C_cyclohexanone_); 3.85 (s, 3H, CH_3_-O), 3.93 (s, 2H, Ar-CH_2_-N), 6.92 (s, 1H, H_Ar_), 7.03 (s, 1H, H_Ar_); 7.14 (d-d, 2H, *J* = 8.8 Hz, H_Ar_), 7,49 (d-d, 2H, *J* = 5.5 Hz, H_Ar_), 7.65 and 7.66 (s, 1H, Ar-CH=C and s, 1H, C=CH-Ar). ^13^C-NMR (100 MHz, CD_3_OD) δ/ppm: 10.9 (2C, CH_3_-CH_2_) 24.0 (1C, C-CH_2_-C_cyclohexanone_), 29.4 and 29.6 (2C, C-CH_2_-C_cyclohexanone_), 47.4 (2C, CH_3_-CH_2_-N-), 56.3 (1C, CH_3_-O), 57.0 (1C, Ar-CH_2_-N), 114.9, 116.3, 116.5, 122.3, 126.4, 133.5, 133.9 (9C, C_Ar_), 136.1, 137.7, 139.3, 139.8 (4C, -C=C-), 153.2 and 163.0 (2C, C_Ar_-O), 165.0 (1C, C_Ar_-F), 191.6 (1C, C=O) [20]. HRESIMS (*m*/*z*) found 422.2178 ([[M − H]]^−^) calculated masses for C_26_H_29_FNO_3_ : 422.2132.

*(2E,6E)-2-({3-[(diethylamino)methyl]-4-hydroxy-5-methoxyphenyl}methylidene)-6-[(4-chlorophenyl)methylidene]cyclohexan-1-one* (**2d**). The compound was an orange powder, in a 76.93% yield, m.p.: 95–97 °C and Rf = 0.45 (ethyl acetate: ethanol = 1:1). FTIR (KBr) υmax cm^−1^: 3032 (CH aromatic), 2972 (CH aliphatic), 1734 (C=O), 1656 (C=C), 1597 and 1491 (C=C aromatic) 1271 (C-N), 1157 (C-O), 839 (C-Cl). ^1^H-NMR (500 MHz, CD_3_OD), δ/ppm: 1.18 (t, 6H, *J* = 7.2 Hz, CH_3_-CH_2_-), 1.78 (m, 2H, C-CH_2_-C_cyclohexanone_); 2.79 (q, 4H, *J* = 7.2 Hz, CH_3_-CH_2_-N), 2.87 and 2.95 (t, 2H, *J* = 5.1 Hz, =C-CH_2_-C_cyclohexanone_ and t, 2H, *J* = 7.3 Hz, =C-CH_2_-C_cyclohexanone_); 3.85 (s, 3H, CH_3_-O), 3.94 (s, 2H, Ar-CH_2_-N), 6.93 (s, 1H, H_Ar_), 7.04 (s, 1H, H_Ar_); 7.42 (d, 2H, *J* = 8.4 Hz, H_Ar_), 7.44 (d, 2H, *J* = 8.7 Hz, H_Ar_), 7.63 and 7.66 (s, 1H, Ar-CH=C and s, 1H, C=CH-Ar). ^13^C-NMR (100 MHz, CD_3_OD) δ/ppm: 11.0 (2C, CH_3_-CH_2_), 24.1 (1C, C-CH_2_-C_cyclohexanone_), 29.2 and 29.6 (2C, C-CH_2_-C_cyclohexanone_), 47.4 (2C, CH_3_-CH_2_-N), 56.3 (1C, CH_3_-O), 56.9 (1C, Ar-CH_2_-N), 114.9, 122.5, 126.5, 129.6, 133.7, 132.8 (9C, C_Ar_), 136.1, 137.7, 138.5, 139.8 (4C, -C=C-), 153.2 and 149.7 (2C, C_Ar_-O), 139.9 (1C, C_Ar_-Cl), 191.6 (1C, C=O) [20]. HRESIMS (*m*/*z*) found 438.1881 ([[M − H]]^−^) calculated masses for C_26_H_29_ClNO_3_ : 438.1837.

*(2E,6E)-2-({3-[(diethylamino)methyl]-4-hydroxy-5-methoxyphenyl}methylidene)-6-[(4-methylphenyl)methylidene]cyclohexan-1-one* (**2e**). The compound was an orange sticky powder, in a 76.9% yield, m.p.: 86–89 °C and Rf = 0.48 (ethyl acetate: ethanol = 1:1). FTIR (KBr) υmax cm^−1^: 3032 (CH aromatic), 2974 (CH aliphatic), 1734 (C=O), 1664 (C=C), 1599 and 1498 (C=C aromatic) 1269 (C-N), 1157 (C-O). ^1^H-NMR (500 MHz, CD_3_OD), δ/ppm: 1.18 (t, 6H, *J* = 7.2 Hz, CH_3_-CH_2_-), 1.77 (m, 2H, C-CH_2_-C_cyclohexanone_), 2.35 (s, 3H, CH_3_-Ar), 2.80 (q, 4H, *J* = 7.2 Hz, CH_3_-CH_2_-N), 2.90 and 2.94 (t, 2H, *J* = 7.3 Hz, =C-CH_2_-C_cyclohexanone_ and t, 2H, *J* = 5.4 Hz, =C-CH_2_-C_cyclohexanone_); 3.85 (s, 3H, CH_3_-O), 3.94 (s, 2H, Ar-CH_2_-N), 6.92 (s, 1H, H_Ar_), 7.04 (s, 1H, H_Ar_); 7.23 (d, 2H, *J* = 7.8 Hz, H_Ar_), 7.37 (d, 2H, *J* = 7,9 Hz, H_Ar_), 7.65 and 7.67 (s, 1H, Ar-CH=C and s, 1H, C=CH-Ar). ^13^C-NMR (100 MHz, CD_3_OD) δ/ppm: 10.9 (2C, CH_3_-CH_2_), 21.4 (1C, CH_3_-Ar), 24.1 (1C, C-CH_2_-C_cyclohexanone_), 29.3 and 29.6 (2C, C-CH_2_-C_cyclohexanone_), 47.5 (2C, CH_3_-CH_2_-N), 54 (1C, CH_3_-O), 56.9 (1C, Ar-CH_2_-N), 114.9, 122.2, 126.3, 126.7, 130.2, 131.5, 134.1 (10C, C_Ar_), 134.3, 137.0, 137.6, 139.5 (4C, -C=C-), 149.7 and 152.9 (2C, C_Ar_-O), 191.6 (1C, C=O) [20]. HRESIMS (*m*/*z*) found 419.1925 ([[M − H]]^−^) calculated masses for C_27_H_32_NO_3_: 419.2384.

### 2.2. Cytotoxicity Test

#### 2.2.1. Brine Shrimp Lethality Test

The assay was carried out according to the principle and protocol previously described by Meyer [23], with slight modification. Artemia salina L. eggs were inserted into a box containing seawater; the box was placed under UV lamp, and after 48 h the eggs hatched into larvae and ready for the test. The compounds (**1a**–**1e**, **2a**–**2e**) were diluted in 10 mL seawater containing 10 larvae (1% DMSO (*v*/*v*)) until concentrations 20, 200, 500, and 1000 ppm were reached. After 24 h, the live and dead shrimp were counted. The mortality rate (%) was obtained by comparing the number of total dead larvae and the total number of larvae. The experiment was conducted in triplicate. The concentrations (dose)-response (% mortality) data were transformed into a straight line using a logit transformation, and the concentration required to kill 50% of the population (LC_50_ values) was derived from the best fit line obtained by linear regression analysis.

#### 2.2.2. MTT Proliferation Assay

The cytotoxic activity of the synthesized compounds was evaluated against human cervix carcinoma (HeLa, ATCC CCL-2) cell lines, human estrogen-dependent breast carcinoma (MCF7, ATCC HTB-22) cell lines, and kidney of an African green monkey (Vero, ATCC CCL-81) as a normal cell lines using the methyl thiazolyl tetrazolium (MTT) method conducted according to the MTT assay protocol published by the American Type Culture Collection (ATCC) [24]. Cisplatin was used as a reference drug. Curcumin was also evaluated as a comparative compound on the tests against Vero and MCF7 cell lines, and doxorubicin was evaluated as an additional comparator on the test against MCF7 cell lines. The assay detects the reduction of yellow tetrazolium (MTT) by metabolically active cells to be purple formazan measured using spectrophotometry [24].

The cells lines were seeded into 96-well plates at a density of 5000 cells per well, replenished with growth media consisting of Dulbecco’s Modified Eagle’s medium (D-MEM) for Vero or Roswell Park Memorial Institute (RPMI) 1640 medium for MCF7 and HeLa, 5% Fetal Bovine Serum (FBS), 100 U/mL penicillin, and 100 μg/mL streptomycin. The cells were incubated at 37 °C in 5% CO_2_ for 24 h. Then, a series concentrations of the tested compounds (1.5, 3.1, 6.2, 12.5, 25.0, 50.0, 100.0, and 200.0 µg/mL) were added to each well of the plate and incubated for 48 h. After that, 10 μL fresh solution of MTT reagent was added to each well, and the plate was incubated in a CO_2_ incubator at 37 °C for 4 h. After the purple precipitate was obtained, the cells were dissolved in ethanol and their optical density was recorded at 595 nm. The experiment was performed in triplicate. Percent proliferation inhibition was calculated using the following formula:

$$\mathrm{Viability}\;\mathrm{cells}\;\mathrm{inhibition}\;\left(\text{\%}\right)=100-\left[\frac{\left(\mathrm{At}-\mathrm{Ab}\right)}{\left(\mathrm{Ac}-\mathrm{Ab}\right)}\right]\;\times 100\%$$

At = absorption of test compound, Ab = absorption of blank, Ac = absorption of control.

The concentration of the synthesized compounds required to inhibit 50% of the growth of the cell lines (IC_50_ values) was calculated by analyzing the relationship between concentrations and percent (%) inhibitions using GraphPad Prism 7 version 7.00 for Windows, GraphPad Software, La Jolla, CA, USA, www.graphpad.com [25].

## 3. Results and Discussion

### 3.1. Chemistry

The novel asymmetrical mono-carbonyl analogs of curcumin (AMACs) (**1a**–**1e**) and its diethylamine Mannich base derivatives (**2a**–**2e**) compounds were synthesized to further explore as anticancer compounds. The synthetic route of the compounds is shown in Scheme 1. The intermediate compounds, (2E)-2-(phenylmethylidene)cyclohexan-1-one and its analogs, were synthesized by the Claisen-Schmidt reaction between benzaldehyde or its analogs with cyclohexanone in the presence of aqueous alkali according to the preparation method of 4-phenylbut-3-en-2-one [19]. The aldol condensation of the intermediate compounds was obtained with vanilin with the addition of diluted HCl/ethanol under reflux conditions for 30 min gave AMACs (**1a**–**1e**). Finally, the Mannich reaction of **1a**–**1e** with diethylamine and formaldehyde under reflux condition in ethanol for 7–11 h (TLC monitoring) afforded the diethylamine Mannich bases of AMACs compounds (**2a**–**2e)**.

The FTIR spectra of compounds **1a**–**1e** showed absorption bands at 3200–3500 cm^−1^ due to the presence of the OH group. The bands at about 1100 cm^−1^ correspond to C-O-C ether, whereas the α,β-unsaturated carbonyl groups of the AMACs were observed as strong bands at about 1,600 cm^−1^. In the ^1^H-NMR spectra, protons of OH phenolic and OCH_3_ group appear as a singlet at δ 5.86–5.90 ppm (1H) and as a singlet at δ 3.91–3.93 ppm (3H), respectively. The two protons of ethenyl chain of the compounds appeared as two singlets at 7.72–7.79 ppm (2H), indicating an asymmetrical compound. The FTIR spectra of compounds **2a**–**2e** showed the disappearance of OH phenolic. The bands at 1151–1271 cm^−1^ correspond to C-O-C and C-N, whereas the α,β-unsaturated carbonyl groups of the compounds were observed as strong bands at 1,734 cm^−1^. In the ^1^H-NMR spectra, protons of the OCH_3_ group appeared as a singlet at δ 3.85 ppm (3H). The two protons of the ethenyl chain of the compounds were observed as two singlets in the range of 7.63 to 7.71 ppm (1H, respectively). The protons of diethylamine groups were observed at 1.16–1.18 as a triplet (6H) and 2.75–2.80 ppm as a quintet (4H), and the protons of methylene adjacent N to the phenyl ring were observed as a singlet (2H) at 3.90–3.95 ppm. The proton signal of OH phenolic disappeared because of exchangeable with deuterium from CD_3_OD used as a solvent in the experiment [20]. Furthermore, the structures of the compounds were supported by ^13^C-NMR and HR-MS, which showed complete agreement with the expected molecular structures.

### 3.2. Cytotoxic Activity

The cytotoxic activity of the compounds was evaluated firstly using a brine shrimp lethality test (BSLT) method as a preliminary test. All the synthesized compounds exhibited toxic activity with LC_50_ value in the range of 29.80–1704.23 µM (13.06–714.49 µg/mL) (Table 1). Compounds **1b**, **2a**, and **2e** had moderate toxicity (LC_50_ value > 100–1000 µg/mL), compound **1a**, **1c**, **1d**, **2b** and **2c** had high toxic activity (LC_50_ > 30–100 µg/mL), and **1e** and **2d** had very high toxic activity (LC_50_ < 30 µg/mL) [26,27]. The BSLT is a rapid, inexpensive, and simple method used to predict the toxicity level of the compounds. The method is not specific to antitumor activity. However, a positive correlation was found between BSLT toxicity and cytotoxicity toward some cell lines [27]. Therefore, in the present study, all the synthesized compounds were followed up to be evaluated for their potential as an anti-cancer agent.

**Figure 1:**
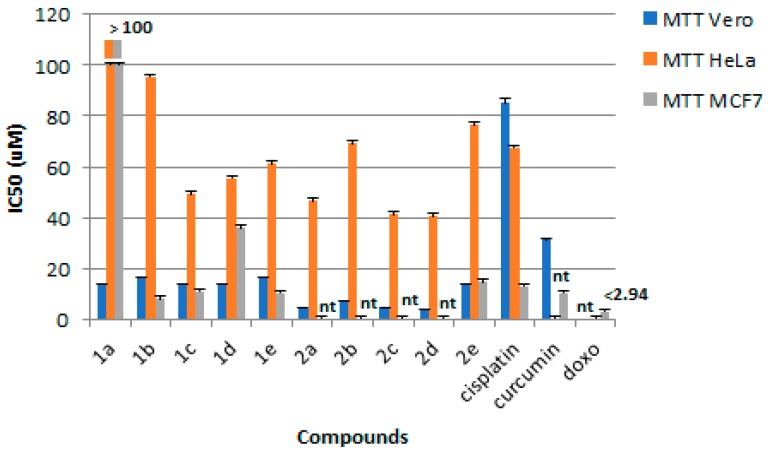
Cytotoxicity of the synthesized compounds against Vero, HeLa, and MCF7 Cell lines. nt = not tested.

The cytotoxic activity of the compounds was evaluated against HeLa and Vero cell lines and for certain selected compounds against MCF7 cell lines. The IC_50_ values and selectivity index (SI) that obtained from the MTT assay are presented in Table 1 and Figure 1. Most of the synthesized compounds (**1b**–**1e** and **2a**–**2e**) exhibited cytotoxic activity against HeLa cell lines with IC_50_ value in the range of 40.65–95.55 µM. In our experiment, cisplatin exhibited an IC_50_ value of 67.59 µM, but an earlier study [28] reported a much lower value (12.3 µM). The reason for this difference could be due to differences in the conditions of the assay [26]. Based on the experimental data, the cytotoxicity of the compound was comparable with that of cisplatin. Unfortunately, all the synthesized compounds exhibited higher cytotoxic activity on Vero cell lines compared to HeLa cell lines (IC_50_ value 3.94–16.15 µM). As a result, the SI of the synthesized compounds was less than 1, indicating that the synthesized compounds were more toxic to a normal cell than to cervix carcinoma cells. Their SI values were lower than that of cisplatin, which had an SI value of 1.26. The results also showed that most of the diethylamine Mannich base derivatives of AMACs (**2a**–**2e**) exhibited slightly higher cytotoxic activity against Hela cell lines than that of the parent compounds (**1a**–**1e**). This result is in line with a previous study that showed that the introduction of Mannich bases enhancing the biological activity of the compounds [29,30,31,32,33]. However, the increasing cytotoxicity was not selective because the effect was also observed at Vero cell lines.

The IC_50_ values of compounds **2a**–**2d** to Vero cell lines were in the range of 3.94–7.28 µM (1.73–3.17 µg/mL). Based on the cytotoxicity criteria of a pure compound (IC_50_ < 4 µg/mL or <10 µM) [34,35], the compounds were considered highly toxic to the normal cell. Therefore, the cytotoxicity of **2a**–**2d** against MCF7 cell lines was not evaluated. The results of the MTT assay against MCF7 cell lines showed that compound **1a** exhibited noncytotoxic activity (IC_50_ > 100 µM), whereas compounds **1b**–**1e** and **2e** exhibited cytotoxic activity with IC_50_ values in the range of 7.86–35.88 µM. Based on the data obtained, the cytotoxic activity of the synthesized compound is more selective to MCF7 cells rather than to HeLa cell lines. Among the synthesized compounds evaluated, compound **1b** was the highest cytotoxic and selective compound against MCF7 cell lines with an IC_50_ value of 7.86 µM (2.75 µg/mL) and an SI value of 1.96. The compound exhibited slightly higher cytotoxic activity (IC_50_ value 7.86 µM) than that of curcumin and cisplatin (IC_50_ values of 10.47 and 12.85 µM, respectively). However, the cytotoxicity was very low when compared with doxorubicin, which exhibits an IC_50_ value <2.94 µM (Table 1, Figure 1). Moreover, the selectivity index (SI) of the compounds to Vero and MCF7 was lower than that of curcumin and cisplatin with SI values of 1.96, 3.00, and 6.61, respectively (Table 1). The greater the SI value, the safer the compound.

The cytotoxicity of compound **1b** containing a 4-OCH_3_ group at the phenyl ring A (Table 1) was higher than **1a**, **1d**, **1c,** and **1e** containing 4-H, 4-Cl, 4-F, and 4-CH_3_, respectively. The results were in line with earlier reported findings that substituent on 4-position of the phenyl ring of the AMACs or MACs significantly influenced the cytotoxicity of the compounds. In addition, a weak electron-donating substitution in 4-position was reported to be the most favorable to the cytotoxic activity of a compound [12,18]. Our data indicate that the electron-withdrawing substitution at 4-position reduced the cytotoxic activity. The effect differs from the electron-withdrawing substitution at 2-position which enhances the cytotoxic activity [12].

As per a standard used earlier, pure compounds are further considered for evaluation as chemotherapeutic agents in preclinical studies using an animal model, should have a potency of 10 µM (4 µg/mL) or less in cell culture studies and an SI value less than 2 [26,35]. Compound **1b** could be considered as a new lead compound for further development to obtain more active and selective chemotherapeutic agents against breast cancer.

**Figure 2:**
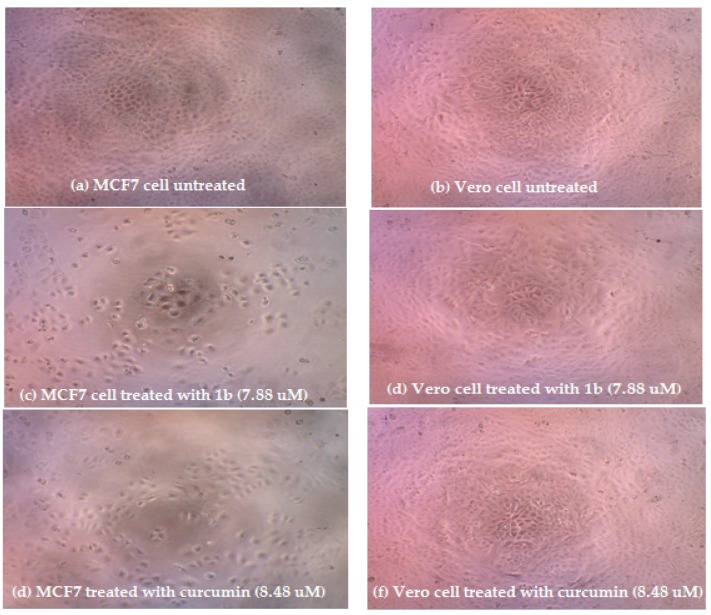

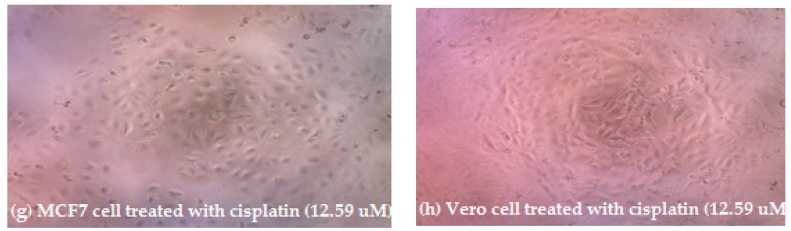
Morphological assessment of MCF7 cells (left) and Vero cells (right) using MTT assay. (**a**,**b**) untreated cells; (**c**,**d**) cells treated with compound **1b** at 7.88 µM; (**e**,**f**) cells treated with curcumin at 8.48 µM; and (**g**,**h**) cells treated with cisplatin at 12.59 µM.

Figure 2 depict the morphological analysis of untreated MCF7 cells (a) and Vero cell (b) versus treated MCF7 and Vero cell with respect to compound **1b** (7.88 µM) (c and d), curcumin (8.48 µM) (e and f), and cisplatin (12.59 µM) (g and h). The figures compare the cytotoxicity of the compounds at the same concentration against human breast cancer cells MCF7 and normal cell Vero.

## 4. Conclusions

A series of asymmetrical mono-carbonyl analogs of curcumin (AMACs) were successfully synthesized. All the synthesized compounds exhibited moderate to very high toxicity based on BSLT, most of them exhibited comparable cytotoxic activity with cisplatin against HeLa cell lines, and the selected compound exhibited moderate to very high cytotoxic activity against MCF7 cell lines. However, all compounds had low SI (<1–1.96). Among the synthesized compounds, compound **1b** showed the highest cytotoxic and selective activity against MCF7 cell lines. This compound could be considered for further development to obtain the more active and selective chemotherapeutic agents against breast cancer.

## Figures and Tables

**Table 1:**
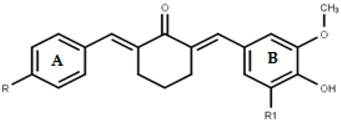
Cytotoxicity of the synthesized compounds against brine shrimp and Vero, Hela, and MCF7 cell lines.

No	Compd	Substuents		LC_50_ (µM)	IC_50_ (µM) ^(^^1)^			SI ^(2)^	
		R_1_	R_2_	BSLT	Vero	Hela	MCF7	Hela	MCF7
1	**1a**	H	H	196.63	13.98 ± 0.04	>100	>100	<1	<1
2	**1b**	OCH_3_	H	322.63	15.43 ± 0.34	95.55 ± 7.19	7.86 ± 1.05	<1	1.96
3	**1c**	F	H	177.36	13.39 ± 0.39	49.15 ± 1.17	10.94 ± 0.79	<1	1.28
4	**1d**	Cl	H	204.09	14.06 ± 0.18	55.60 ± 1.49	35.88 ± 4.57	<1	<1
5	**1e**	CH_3_	H	78.71	16.15 ± 0.18	61.19 ± 2.86	10.39 ± 0.36	<1	1.55
6	**2a**	H	X	921.08	4.14 ± 0.21	46.61 ± 1.54	nt	<1	-
7	**2b**	OCH_3_	X	80.21	7.29 ± 0.12	69.29 ± 3.17	nt	<1	-
8	**2c**	F	X	88.37	4.23 ± 0.32	41.10 ± 0.16	nt	<1	-
9	**2d**	Cl	X	29.80	3.94 ± 0.07	40.65 ± 0.98	nt	<1	-
10	**2e**	CH_3_	X	1704.23	15.02 ± 0.14	76.61 ± 4.27	14.55 ± 1.96	<1	1.03
11	Curcumin	-	-	nt	31.41 ± 0.41	nt	10.47 ± 1.10	-	3.00
12	Cisplatin	-	-	nt	84.66 ± 2.09	67.59 ± 2.04	12.85 ± 1.35	1.26	6.61
13	Doxorubicin	-	-	nt	nt	nt	< 2.94	-	-

^(1)^ Values are the mean ± SD (*n* = 3); nt = not tested. ^(2)^ SI = Selectivity Index = IC_50_ value normal cell/IC_50_ value cancer cell. X = CH_2_-N(CH_2_-CH_3_)_2_.
